# Data on greenhouse gas fluxes from smallholder cropping systems in Upper Eastern Kenya

**DOI:** 10.1016/j.dib.2025.111955

**Published:** 2025-08-05

**Authors:** Collins M. Musafiri, Shaankua E. Lemarpe, Milka N. Kiboi, Felix K. Ngetich

**Affiliations:** a Cortile Scientific Limited, PO Box 34991, 00100, Nairobi, Kenya; b Research Centre for Smallholder Farmers, PO Box 10451, 30100 Eldoret, Kenya; c Department of International Cooperation, Research Institute of Organic Agriculture (FiBL), Ackerstrasse 113, 5070 Frick, Switzerland; d School of Agricultural and Food Sciences, Jaramogi Oginga Odinga University of Science and Technology (JOOUST), PO Box 210, 40601, Bondo, Kenya

**Keywords:** Carbon dioxide, Methane, Nitrous oxide, Smallholder farms, Cropping systems, Yield-scaled emissions

## Abstract

This dataset presents greenhouse gas (GHG) fluxes—carbon dioxide (CO₂), methane (CH₄), and nitrous oxide (N₂O) measurements collected from smallholder cropping systems in Chuka, Upper Eastern Kenya. The study aimed to assess the effects of different cropping systems on soil GHG fluxes, as described in a related study (Lemarpe et al., 2023). Data were collected over two cropping seasons from five cropping systems: sole maize, maize-bean intercrop, agroforestry, banana and coffee replicated three times. The study used static chambers to measure GHG fluxes, which were then analyzed using gas chromatography. The dataset also consists of soil physicochemical parameters (bulk density, total nitrogen, organic carbon, and pH), soil moisture, rainfall, and crop yield data for maize and beans. This dataset offers findings on how agricultural practices affect GHG emissions and the possibility of smallholder cropping systems, such as agroforestry, to act as nature-based solutions for climate change mitigation.

Specifcations Table

**Table:** 

Subject	Agricultural Sciences
Specific subject area	Agronomy, Environmental Science, Soil Science
Type of data	Tables Raw
Data collection	Field experiments using static chamber methods and gas chromatography (GC) analysis
Data source location	Chuka, Tharaka-Nithi County, Upper Eastern Kenya
Data accessibility	Repository name: Mendeley Title of the dataset: Datasets for the article "Smallholder cropping systems contribute limited greenhouse gas fluxes in upper Eastern Kenya" Direct URL to data: https://data.mendeley.com/datasets/72b3hyjgdh/3
Related research article	Lemarpe, S.E., Musafiri, C.M., Kiboi, M.N., Ng'etich, O.K., Macharia, J.M., Shisanya, C.A., Kibet, E., Zeila, A., Mutuo, P. and Ngetich, F.K., 2023. Smallholder cropping systems contribute limited greenhouse gas fluxes in upper Eastern Kenya. Nature-Based Solutions, 4, p.100098. https://doi.org/10.1016/j.nbsj.2023.100098 [1]

## 1 Value of the Data

• These data provide vital knowledge about how smallholder farming operations affect greenhouse gas emissions in sub-Saharan Africa.
• The dataset is valuable for agricultural researchers, policymakers, and climate change modelers interested in nature-based solutions and sustainable agriculture.
• The dataset provides a basis for comparing greenhouse gas (GHG) fluxes occurring in various cropping systems to establish optimal agricultural practices for reducing climate change.
• The data provide essential information to measure agroforestry's dual capability as a sustainable method to control GHG emissions alongside continued agricultural yield levels.

## 2 Background

Agricultural systems play a key role in contributing to and mitigating climate change. The agricultural sector contributes 11–14 % of anthropogenic GHG emissions, while land-use changes account for an additional 17 % [2]. Smallholder farms, which control 80 % of agricultural land in sub-Saharan Africa (SSA), need nature-based solutions given their vulnerability to resource constraints and limited adaptive capacity in changing temperatures and altered rainfall patterns. Climate variability creates two significant impacts on agriculture since it decreases food security and disrupts farming systems, thus necessitating the implementation of natural approaches [3,4]. The literature reveals GHG emissions have not been extensively studied in SSA [[5], [6], [7]–8]. This study investigated the extent to which different cropping systems emit GHGs, demonstrating that nature-based smallholder farming practices can mitigate GHG emissions while improving farm productivity

## 3 Data Description

This dataset contains GHGs flux measurements and associated data collected from smallholder farming systems in Chuka, Upper Eastern Kenya. The dataset focuses on two cropping seasons, namely the Long Rain 2019 (LR 2019) and Short Rain 2019 (SR 2019). Data were collected between 30th April 2019 and 4th February 2020, with the Long Rain (LR) season occurring from 30th April to 30th June 2019 and the Short Rain (SR) season from 15th October to 31st December 2019. Dry period occurred from 1st July to 14th October 2019 and from 1st January to 4th February 2020, with minimal rainfall during these times. The dataset also includes additional data such as soil properties, rainfall measurements, soil water content, and crop yields across five distinct cropping systems: sole maize, maize-bean intercrop, agroforestry, banana and coffee. The data was collected from two smallholder farms, with each cropping system being replicated three times for consistency and reliability.

The data is distributed across six distinct sheets. These sheets address different aspects of the study, including site-specific details, GHGs flux measurements, weather patterns, soil properties, soil moisture content, and crop yields. The first sheet, Metadata, provides background information on the study, including the site name, geographical coordinates, study duration, and data ownership. The GHG Fluxes sheet includes each cropping system's measured CH_4_, N_2_O, and CO_2_ emissions, recorded at regular intervals. The Rainfall sheet presents daily precipitation data collected using a manual rain gauge at Kangutu Primary School. The Soil Properties sheet contains data on key soil characteristics such as bulk density, pH, total nitrogen, and soil organic carbon ( %) and Carbon Nitrogen Ratio. The Soil Water Content sheet reports volumetric soil moisture content, and the Biomass/Yield Data sheet tracks the biomass yields for different crops (maize and beans) under each cropping system.

The temporal resolution of the datasets varies depending on the type of data. The GHG fluxes were measured following key events such as rainfall, land preparation, planting, weeding and harvesting. Data were collected weekly during the wet seasons and bi-weekly during the dry season. Rainfall data were collected daily, providing valuable information on precipitation patterns for the study. Soil properties were measured at two points: once at the beginning of the Long Rain season and again at the end of the Short Rain season. Soil water content was recorded during GHG fluxes measurements, and biomass yield data were collected at the end of each cropping season (LR 2019 and SR 2019).

The data is organized by plot, cropping system, and season. Each entry includes identifiers for the farm (Farm 1 or Farm 2), the treatment (specific cropping system), and the sampling date. This structure enables tracking changes over time and comparing data across different treatments, seasons and plots (replicates).

## 4 Experimental Design, Materials and Methods

### 4.1 Experimental design

Field experiments were conducted on two smallholder farms (37.6833° S, 0.3378°E and 37.6833° S, 0.3491° E) in Kangutu sub-location, Chuka/Igamba Ng'ombe sub-county, Tharaka-Nithi County, Upper Eastern Kenya. The research sites lie within the Upper Midland 3 (UM3) agroecological zone characterized by rain-fed agricultural systems, predominantly growing maize, beans, coffee, and bananas, with agroforestry practices present in some farms. The experiment included five cropping systems: (i) sole maize, (ii) maize–bean intercrop, (iii) agroforestry, (iv) banana, and (v) coffee. The cropping systems were replicated three times in each farm. The selection of farms and cropping systems aimed at reflecting typical smallholder farming practices.

### 4.2 Greenhouse gas measurement

Soil GHG fluxes were measured using the static chamber method. Each cropping system had three chambers installed randomly within and between crop rows for the entire experimental period. Round chambers with a radius of 0.2 m and a height of 0.1 m consisting of two parts (base and lid) were used. During sampling events, gas samples were collected at intervals of 0, 10, 20, and 30 min. The GHGs flux sampling was conducted weekly during the wet season, bi-weekly during the dry season, and following major agricultural activities such as land preparation, planting, input application, weeding, and harvesting, as well as after each rainfall event to capture variations in emissions influenced by these factors. Samples were analyzed using Gas Chromatography (GC) equipped with a ⁶³Ni-Electron Capture Detector (ECD) for N₂O analysis and a Flame Ionization Detector (FID) for CH₄ and CO₂. The GHG fluxes were calculated using linear regression of gas concentrations against time intervals (chamber closure duration). Data quality was assessed, and samples with low linearity (R²<0.90) for CO₂ were excluded.

### 4.3 Soil sampling and analysis

Soil samples were collected from the topsoil (0–20 cm depth) at each cropping system at the baseline and end-line of the experiments. The samples were analyzed for bulk density, total nitrogen (N), Soil organic carbon (SOC), and pH. Bulk density was determined from undisturbed soil cores and analyzed gravimetrically, while total N and SOC were measured using a C/ N elemental analyzer. Soil moisture was assessed gravimetrically and converted to volumetric units. Precipitation data were collected using a manual rain gauge installed at Kangutu Primary School.

### 4.4 Biomass and yield measurements

Crop yield (bean and maize) was harvested manually from designated sub-plots (2 m x 2 m) within each cropping system. Fresh biomass was separated into grains, leaves, stems, and roots, and it was weighed, air-dried, and re-weighed to determine dry weight. The Season column in the biomass data refers to the two distinct rainfall periods in the study area, which experience a bimodal rainfall pattern. These periods are classified as Long Rain and Short Rain. The Long Rain typically occurs from March to May, while the Short Rain occurs from October to December. The data includes measurements taken during Short Rain 2019 and Long Rain 2019 to account for variations in biomass and greenhouse gas emissions associated with different rainfall seasons. Grain yields were reported at a standardized moisture content (12.5 %).

## Limitations

This study has several limitations, including using only two farms, which limits the generalizability of results. The dataset covers only two seasons (Long Rain 2019 and Short Rain 2019), which may not capture long-term trends. The COVID-19 disruptions affected sampling consistency, while dynamic farmer activities and fixed chamber measurements may have missed peak emissions. Additionally, chamber installation may have altered soil conditions. These factors should be considered when interpreting the data.

## Ethics Statement

The authors have read and followed the ethical requirements for publication in Data in Brief and confirm that the current work does not involve human subjects, animal experiments, or any data collected from social media platforms.

## Declaration of Generative Ai and Ai-Assisted Technologies in the Writing Process

During the preparation of this work, the authors used ChatGPT to improve language and readability. After using this tool/service, the authors reviewed and edited the content as needed and take(s) full responsibility for the content of the publication.

## Data Availability

Mendeley DataDatasets for the article “Smallholder cropping systems contribute limited greenhouse gas fluxes in upper Eastern Kenya” (Original data).
